# Chatbot assistance in precision oncology treatment decision-making

**DOI:** 10.1093/oncolo/oyaf316

**Published:** 2025-10-12

**Authors:** Hannah Burnette, Kylie Fletcher, Christine Micheel, Ben Ho Park, Daniel H Johnson, John Cole, Kevan Simms, Marc Matrana, Douglas B Johnson

**Affiliations:** Vanderbilt University Medical Center and Vanderbilt Ingram Cancer Center, Nashville, TN 37232, United States; Vanderbilt University School of Medicine, Nashville, TN 37232, United States; Vanderbilt University Medical Center and Vanderbilt Ingram Cancer Center, Nashville, TN 37232, United States; Vanderbilt University Medical Center and Vanderbilt Ingram Cancer Center, Nashville, TN 37232, United States; Ochsner Cancer Institute, New Orleans, LA 70115, United States; Ochsner Cancer Institute, New Orleans, LA 70115, United States; Ochsner Cancer Institute, New Orleans, LA 70115, United States; Ochsner Cancer Institute, New Orleans, LA 70115, United States; Vanderbilt University Medical Center and Vanderbilt Ingram Cancer Center, Nashville, TN 37232, United States

**Keywords:** precision oncology, chatbot, ChatGPT

## Abstract

Artificial intelligence chatbots have shown promise in medical settings, but their ability to interpret complex molecular data is not clear. Here, we assessed 50 different patient scenarios with clinical and molecular data and found that chatbots provided mostly accurate and comprehensive recommendations, although key treatment options were omitted occasionally, and non-data driven treatments were recommended in cases with multiple mutations.

## Introduction

The advent of artificial intelligence chatbots is rapidly transforming healthcare. With the ability to generate human-like speech using large language models (LLMs), chatbots can respond to medical queries,^1^ potentially including those in the realm of precision oncology.

Cancer molecular profiling produces a vast array of data, which may be complex for physicians to interpret and determine actionability of the findings. Molecular tumor boards (MTB) are one avenue where results are reviewed with a team of experts.^2^ However, most clinicians do not have access to such resources, and these forums are difficult to scale. Early results suggest that LLMs are able to provide reasonable treatment options even in complex cancer scenarios though with important limitations.^3–8^ Thus, to assess whether chatbots could provide useful input to molecular profiling results, we devised scenarios that physicians might encounter in a clinical setting to assess chatbot responses for accuracy and completeness. Scenarios were “straightforward” (approved drug and well-validated mutation), “ambiguous” (molecular alteration with an approved drug in a different cancer, or alteration with a limited data for an approved therapy), or “MTB-like” (where a case was de-identified and taken from an actual MTB presentation).

## Methods

We designed 25 straightforward, 15 ambiguous, and 10 MTB-like scenarios (see Supplementary material).

### Scenario formulation

One physician (DBJ) generated 40 clinical scenarios with cancer and molecular information. These were defined as “straightforward,” with a molecular alteration corresponding to an approved molecularly-targeted agent in that tumor type (eg *BRAF*^V600E^ mutated melanoma), or as “ambiguous” with a molecular alteration without a corresponding approval (eg *BRAF*^K601E^ mutated melanoma). An additional 10 “Molecular tumor board-like” (MTB-like) scenarios were generated based on real cases at VUMC. The clinical scenario (with altered patient age) along with the 1-2 most salient mutations were included, with the remainder of the molecular profile pulled from relevant tumor types in cBio Portal^10^ to demonstrate the molecular complexity of pre-treated tumors and shield patient identity. Scenarios were entered into ChatGPT (version 4) with the following prompt: “Please assume you are an expert in precision oncology giving recommendations to an oncologist. Please assume no prior treatment has been given unless specified. In your answer, please list (1) any treatments that the molecular or genetic testing results may suggest and their expected effectiveness, and (2) any additional treatment options besides those suggested by the testing results.” Cases scoring <3 on accuracy were rescored using two different engines (ChatGPT version 4o and Google Gemini Flash version 1.5).

### Response scoring

Chatbot responses were provided to two physicians (DBJ and MM, both directors of precision oncology at their centers) with a third physician (BP) for cases scoring <3 for accuracy, who rated using a 3-point Likert scale for accuracy (scores of 1—inaccurate, 2—partly accurate, 3—completely accurate) and completeness (1—incomplete, 2—appropriate alternatives partially listed, 3—appropriate alternatives listed). Ambiguous scenarios were also rated on confidence (1—presented only targeted therapy with confidence and no alternatives, 2—overconfident in targeted therapy, 3—appropriate confidence). Medians and means were tabulated; categories were compared using Mann-Whitney U testing between straightforward and ambiguous scenarios.

## Results

Overall, scores were high for all categories of answers. Table 1 shows the mean/median accuracy and completeness ratings for each scenario type. There was no difference in average scores between straightforward and ambiguous scenarios (*P *= .99). Similarly, there was no difference in completeness for types of scenarios (*P *= .70). We rated confidence for the ambiguous scenarios (eg whether the engine overweighted the likelihood of response). The average rating for confidence was 2.87 (median 3) for both reviewers. Data supplement provides scenarios, scores, and reasons for scores less than 3.

**Table 1.** Scores for reviewers by scenario type.

Scenario	N	Mean accuracy score (median): Reviewer 1	Mean completeness score (median): Reviewer 1	Mean accuracy score (median): Reviewer 2	Mean completeness score (median): Reviewer 2
**Straightforward**	25	2.92 (3)	2.76 (3)	2.92 (3)	3.00 (3)
**Ambiguous**	15	2.87 (3)	3.00 (3)	3.00 (3)	2.87 (3)
**MTB-like**	10	2.30 (2)	2.90 (3)	2.90 (3)	3.00 (3)
Re-graded—ChatGPT v4o*	11	2.61 (3)	2.70 (3)		
Re-graded—Gemini Flash v1.5*	11	2.58 (3)	2.52 (3)		

* Mean/median scores graded by all three reviewers.

Answers scoring <3 by either Reviewer were assessed by newer versions (ChatGPT version 4o and Google Gemini Flash version 1.5) and regraded by three reviewers (2 straightforward questions, 3 ambiguous, and 6 MTB-like). Mean accuracy score was 2.61 (median 3) and completeness was 2.70 (median 3) for ChatGPT v4o; mean accuracy was 2.58 and completeness 2.52 for Google Gemini Flash v1.5 (median 3 for each); scores were similar for all three reviewers (data not shown). These were comparable to the original scores using ChatGPT v4 for these questions (mean/median 2.5 for accuracy and 2.91/3 for completeness).

## Discussion

This study found that chatbots provided fairly accurate and complete answers when presented scenarios of patients with cancer with a diverse range of molecular findings. The engine often suggested appropriate answers and provided alternative, non-molecularly-guided cancer treatments (eg both BRAF/MEK inhibition and immunotherapy for *BRAF* mutated melanoma). Further, it was generally able to parse actionable from non-actionable molecular alterations. Despite this, it missed some appropriate treatment options (eg elacestrant for *ESR1* mutation following endocrine therapy), and proposed some therapies without strong evidence bases, particularly in MTB-like scenarios. No obvious improvement was observed with newer engines although definitive comparisons are difficult with small sample size.

Limitations include using a single prompt (vs. prompt engineering, which could increase accuracy), potential for lack of reproducibility (with different prompts or chatbot versions), lack of a gold standard scoring system for accuracy/completeness, and updated versions of each chatbot even since study design and conduct. This study shows potential for ChatGPT and Google Gemini to provide guidance to providers with complex molecular profiling information. Other chatbots may also hold promise in this area; additional training on molecular tumor board reports, oncology knowledge-bases, regulatory approvals, and oncology guidelines could further enhance this capacity.^9^ Specifically designed LLMs trained for interpreting molecular data and integrating real-time updates and clinical trial availability will likely be a future state. Currently available chatbots may also be to supplement molecular tumor boards where a team of experts provides recommendations based on molecular profiling, and may serve as a useful adjunct to avoid missing potentially helpful therapies. Clinicians will need to carefully filter information though (perhaps in part by querying the specific evidence backing up a recommendation) prior to incorporating treatment advice. Ultimately, the use of chatbots should not supplant, but rather supplement, human expert interpretation and input.

## Supplementary Material

oyaf316_Supplementary_Data

## Data Availability

All data are included in the supplement.
