# Supplementary material for: Chatbot assistance in precision oncology treatment decision-making
Source: Oncologist. 2025 Oct 12;30(10):oyaf316. doi: 10.1093/oncolo/oyaf316 (PMC12517745; doi:10.1093/oncolo/oyaf316)
Supplement: oyaf316_Supplementary_Data [file oyaf316_supplementary_data.zip › Supplemental Information_Scenario Examples.docx]

| **Supplemental Information: Scenario Examples**  **NOTE: All scenarios followed by: Please assume you are an expert in precision oncology giving recommendations to an oncologist. Please assume no prior treatment has been given unless specified. In your answer, please list 1) any treatments that the molecular or genetic testing results may suggest and their expected effectiveness, and 2) any additional treatment options besides those suggested by the testing results.**  **All other scenarios, generated answers, and scores not listed here are available upon request.** |
| --- |

| **Clear Scenario Example – ChatGPT v4.0** | | | | | | | |
| --- | --- | --- | --- | --- | --- | --- | --- |
| **Age** | **Disease Type/Stage/Metastasis** | **Prior Treatments** | **Mutations** | **Rater #** | **Accuracy** | **Completeness** | **Confidence** |
| 45 | Melanoma/IV/Lung/Liver | None | BRAF V600E | **1** | 3 | 3 |  |
|  |  |  |  | **2** | 3 | 3 |  |
| **Example of Output:** | | | | | | | |
| ### 1) Treatments Suggested by Molecular/Genetic Testing Results:  #### Targeted Therapy:  - **BRAF Inhibitors (Vemurafenib, Dabrafenib):** These drugs specifically target the BRAF V600E mutation and have shown significant effectiveness in shrinking melanoma tumors and slowing disease progression.  - **MEK Inhibitors (Trametinib, Cobimetinib):** Often used in combination with BRAF inhibitors to enhance effectiveness and overcome resistance. The combination is more effective than BRAF inhibition alone in BRAF-mutant melanoma.  **Expected Effectiveness:**  - The combination of BRAF and MEK inhibitors has been shown to improve survival rates significantly in patients with metastatic melanoma harboring the BRAF V600 mutation. Clinical trials have demonstrated improved overall survival, progression-free survival, and higher response rates compared to chemotherapy or BRAF inhibitors alone.  ### 2) Additional Treatment Options:  #### Immunotherapy:  - **Checkpoint Inhibitors (Nivolumab, Pembrolizumab, Ipilimumab):** These drugs work by unleashing the immune system to attack cancer cells. Nivolumab and pembrolizumab target PD-1, while ipilimumab targets CTLA-4. They can be used alone or in combination depending on the patient's overall health and disease characteristics.  **Expected Effectiveness:**  - Immunotherapy has been a breakthrough in melanoma treatment, especially for advanced stages. It has shown durable responses and improved survival rates in a subset of patients. However, the effectiveness varies widely among individuals, and not all patients will respond.  #### Chemotherapy:  - While less commonly used due to the effectiveness of targeted therapies and immunotherapies, chemotherapy (such as dacarbazine) may still be an option, particularly in settings where other treatments are not available or suitable.  #### Clinical Trials:  - Enrolling in clinical trials for new drugs or combinations that are not yet widely available can be a valuable option for some patients, offering access to cutting-edge treatments.  #### Supportive Care:  - It's also important to address symptoms and improve the quality of life through supportive care, including pain management, nutritional support, and psychological counseling.  ### Monitoring and Adjustments:  Treatment plans should be regularly reviewed and adjusted based on the response to treatment, side effects experienced, and any changes in the patient's overall health. It's also crucial to monitor for resistance to targeted therapies and consider switching treatment strategies if resistance develops.  This recommendation is based on the current standard of care and available evidence. The actual choice of treatment should be individualized based on the patient's specific clinical scenario, including comorbidities, preferences, and potential side effects. | | | | | | | |

| **MTB-Like Scenario Example - ChatGPT v4.0** | | | | | | | |
| --- | --- | --- | --- | --- | --- | --- | --- |
| **Age** | **Disease Type/Stage/Metastasis** | **Prior Treatments** | **Mutations** | **Rater #** | **Accuracy** | **Completeness** | **Confidence** |
| 60 | Endometrial/NA/NA | Carboplatin and Paclitaxel, Letrozole, and Lenvatinib/Pembrolizumab | PIK3CA E542K, ARID1A L649Sfs*9, CTNNB1 S33F | **1** | 2 | 2 | 3 |
|  |  |  |  | **2** | 3 | 3 | 3 |
| **Example of Output:** | | | | | | | |
| ### ### Treatment Recommendations for a 60-Year-Old Woman with Endometrial Cancer and Specific Genetic Alterations  #### Molecular and Genetic Testing Results:  1. **PIK3CA E542K Mutation**  2. **ARID1A L649Sfs*9 Mutation**  3. **CTNNB1 S33F Mutation**  #### 1. Treatments Suggested by Molecular or Genetic Testing:  **1.1. PIK3CA Mutation:**  - **Alpelisib (Piqray):**  - **Rationale:** Alpelisib is a PI3K inhibitor specifically targeting the alpha isoform, which is implicated in the PIK3CA mutation.  - **Expected Effectiveness:** Studies in breast cancer with PIK3CA mutations have shown effectiveness; however, evidence in endometrial cancer is emerging. Alpelisib in combination with hormonal therapy (e.g., letrozole) could be considered if the patient is hormone receptor-positive.  **1.2. ARID1A Mutation:**  - **EZH2 Inhibitors (e.g., Tazemetostat):**  - **Rationale:** ARID1A mutations often lead to dependency on EZH2, making EZH2 inhibitors a potential targeted therapy.  - **Expected Effectiveness:** Clinical trials have shown promising results in ARID1A-mutant tumors, including endometrial cancer.  **1.3. CTNNB1 Mutation:**  - **Wnt/β-catenin Pathway Inhibitors:**  - **Rationale:** CTNNB1 mutations lead to activation of the Wnt/β-catenin pathway. Targeting this pathway could be beneficial.  - **Expected Effectiveness:** Specific inhibitors are still under investigation, but targeting this pathway may help manage the progression of tumors harboring CTNNB1 mutations.  #### 2. Additional Treatment Options:  **2.1. Clinical Trials:**  - **Recommendation:** Enrollment in clinical trials focusing on novel therapies for endometrial cancer or targeting specific genetic mutations (e.g., PIK3CA, ARID1A, CTNNB1) should be considered. Clinical trials often provide access to cutting-edge treatments and are a valuable option for patients with advanced cancer.  **2.2. Immune Checkpoint Inhibitors:**  - **Agents:** Pembrolizumab (Keytruda) or Nivolumab (Opdivo) if not previously used.  - **Rationale:** Immune checkpoint inhibitors have shown efficacy in various cancers, including endometrial cancer, particularly in those with high microsatellite instability (MSI-H) or mismatch repair deficiency (dMMR).  - **Expected Effectiveness:** Efficacy may vary; however, durable responses have been observed in some patients with endometrial cancer.  **2.3. Combination Therapies:**  - **Example:** Combining targeted therapies with immune checkpoint inhibitors (e.g., Alpelisib plus Pembrolizumab).  - **Rationale:** Synergistic effects may enhance treatment efficacy by targeting multiple pathways.  - **Expected Effectiveness:** Combination therapies are actively being studied and may offer improved outcomes.  **2.4. Hormonal Therapy:**  - **Agents:** Megestrol acetate or Tamoxifen in cases where hormonal receptors are positive.  - **Rationale:** Hormonal therapy can be effective in hormone receptor-positive endometrial cancer.  - **Expected Effectiveness:** Varies based on hormone receptor status and previous treatments.  **2.5. Supportive Care:**  - **Recommendation:** Comprehensive supportive care to manage symptoms and improve quality of life, including pain management, nutritional support, and psychosocial care.  ### Summary:  The molecular testing results suggest the use of targeted therapies such as Alpelisib for the PIK3CA mutation and EZH2 inhibitors for the ARID1A mutation. Additionally, clinical trials, immune checkpoint inhibitors, and combination therapies should be considered to manage this patient's advanced endometrial cancer. Supportive care remains essential throughout treatment.  Regular follow-up and re-evaluation of treatment response and tolerance are crucial to optimize the patient's management plan. | | | | | | | |

| **Ambiguous Scenario Example – ChatGPT v4o** | | | | | | | |
| --- | --- | --- | --- | --- | --- | --- | --- |
| **Age** | **Disease Type/Stage/Metastasis** | **Prior Treatments** | **Mutations** | **Rater #** | **Accuracy** | **Completeness** | **Confidence** |
| 80 | NSCLC/NA/NA | None | ROS1 W712 truncating | **1** | 3 | 3 | 3 |
|  |  |  |  | **2** | 3 | 3 | 3 |
|  |  |  |  | **3** | 3 | 3 | 3 |
| **Example of Output:** | | | | | | | |
| For an **80-year-old man with non-small cell lung cancer (NSCLC, adenocarcinoma subtype)** harboring a **ROS1 W712* truncating mutation**, here are evidence-based recommendations:  ### **1. Treatments Suggested by Molecular or Genetic Testing (ROS1 W712* Mutation)**  The **ROS1 W712*** truncating mutation is a rare alteration that results in loss of the kinase domain function. Unlike ROS1 fusions, which are sensitive to ROS1 tyrosine kinase inhibitors (TKIs), truncating mutations are unlikely to respond to standard ROS1-targeted therapies. Instead, therapeutic strategies should focus on alternative mechanisms and broader tumor control.  #### A. **Immune Checkpoint Inhibitors**  1. **Pembrolizumab (Keytruda) Monotherapy (if PD-L1 ≥50%)**  - **Mechanism:** Anti-PD-1 immune checkpoint blockade enhances the immune system's ability to target cancer cells.  - **Expected Effectiveness:**  - In PD-L1-high NSCLC, pembrolizumab monotherapy achieves:  - Objective response rate (ORR): ~45%.  - Median overall survival (OS): ~20 months.  - **Considerations:** Preferred in patients with high PD-L1 expression and no contraindications to immunotherapy.  2. **Combination Immunotherapy + Chemotherapy (if PD-L1 <50% or Tumor Burden is High):**  - **Regimen:** Pembrolizumab + platinum doublet chemotherapy (e.g., carboplatin + pemetrexed for nonsquamous NSCLC).  - **Expected Effectiveness:**  - Based on the KEYNOTE-189 trial:  - Median OS: ~22 months.  - ORR: ~48%.  - **Considerations:** Synergistic effects in tumors with lower PD-L1 expression or aggressive disease.  #### B. **ROS1-Directed Therapy (Experimental/Off-Label)**  - **Entrectinib or Crizotinib:**  - These ROS1 TKIs are highly effective for **ROS1 fusions** but unlikely to be effective for truncating mutations like W712*.  - Use only if additional testing reveals a co-occurring ROS1 fusion or activating mutation.  ### **2. Additional Treatment Options Beyond ROS1-Specific Therapies**  #### A. **Platinum-Based Chemotherapy**  - **Carboplatin + Pemetrexed:**  - Standard regimen for advanced nonsquamous NSCLC.  - **Expected Effectiveness:** Effective in patients without actionable oncogenic drivers.  - Maintenance pemetrexed can prolong progression-free survival (PFS) after induction therapy.  #### B. **Combination Immunotherapy + VEGF Inhibitor**  - **Atezolizumab + Bevacizumab + Carboplatin + Paclitaxel:**  - **Mechanism:** Immune checkpoint blockade with VEGF inhibition and chemotherapy.  - **Expected Effectiveness:**  - IMpower150 trial demonstrated OS benefit, even in patients with comorbidities or poor performance status.  - **Considerations:** Option for patients who can tolerate combination therapy.  #### C. **Clinical Trials**  - **ROS1 Truncation-Specific Research:**  - Explore clinical trials targeting rare ROS1 mutations or alternative resistance mechanisms.  - **Novel Immunotherapy Combinations:**  - Investigate agents targeting immune checkpoints beyond PD-1/PD-L1, such as LAG-3 or TIGIT.  #### D. **Radiotherapy**  - Palliative radiotherapy for symptomatic metastases (e.g., bone or liver metastases).  ### **3. Other Considerations**  #### A. **Molecular Profiling**  - Ensure comprehensive genomic testing to rule out other actionable mutations (e.g., MET, RET, or NTRK) or co-alterations in the tumor.  #### B. **Age and Comorbidities**  - Given the patient’s advanced age, consider tolerability and potential side effects of systemic therapies when selecting the treatment regimen.  #### C. **Supportive Care**  - Focus on symptom management, including nutritional support and interventions for fatigue or pain related to metastatic disease.  ### **Management Summary**  1. **Primary Therapy:**  - **Pembrolizumab monotherapy** (if PD-L1 ≥50%) or **pembrolizumab + platinum chemotherapy** for lower PD-L1 expression or higher tumor burden.  2. **Subsequent Options:**  - Platinum-based chemotherapy (e.g., carboplatin + pemetrexed) or the **atezolizumab + bevacizumab combination** if immune checkpoint inhibitors are insufficient or contraindicated.  3. **Clinical Trials:**  - Investigate novel therapies targeting ROS1 truncations or rare NSCLC mutations.  4. **Supportive Care:** Ensure optimal symptom management and quality of life. | | | | | | | |

| **Clear Scenario Example – Google Gemini 1.5 Flash** | | | | | | | |
| --- | --- | --- | --- | --- | --- | --- | --- |
| **Age** | **Disease Type/Stage/Metastasis** | **Prior Treatments** | **Mutations** | **Rater #** | **Accuracy** | **Completeness** | **Confidence** |
| 57 | ER/PR+ HER2- Breast Cancer/NA/NA | Three regimens containing aromatase inhibitors | ESR1 | **1** | 3 | 3 |  |
|  |  |  |  | **2** | 3 | 3 |  |
|  |  |  |  | **3** | 3 | 2 |  |
| **Example of Output:** | | | | | | | |
| This is for informational purposes only. For medical advice or diagnosis, consult a professional.  **1. Treatments Suggested by Molecular/Genetic Testing:**  * **Selective Estrogen Receptor Degraders (SERDs):**  * **Elacestrant (Orserdu):** This is an FDA-approved SERD specifically for postmenopausal women with ER+/HER2- breast cancer with ESR1 mutations who have progressed on at least one line of endocrine therapy. Elacestrant has shown significant clinical benefit in this patient population.  * **Fulvestrant:** Another SERD that may be considered, although elacestrant is generally preferred in the presence of ESR1 mutations.  * **Combinations:**  * **SERD + CDK 4/6 inhibitors (palbociclib, ribociclib, abemaciclib):** Combining a SERD with a CDK 4/6 inhibitor may further improve outcomes in patients with ESR1-mutated breast cancer.  **2. Additional Treatment Options:**  * **Chemotherapy:**  * **Taxanes (paclitaxel, docetaxel):** May be considered, especially if not previously used.  * **Anthracyclines (doxorubicin, epirubicin):** May be considered, especially if not previously used.  * **Immunotherapy:**  * **Immune Checkpoint Inhibitors (ICIs):** While less common in ER-positive breast cancer, some patients may benefit from ICIs such as pembrolizumab or atezolizumab, especially if they have specific tumor markers (e.g., high PD-L1 expression).  * **Clinical Trials:**  * Participation in clinical trials investigating novel therapies for ESR1-mutated breast cancer is strongly encouraged. These trials may offer access to the latest and most promising treatments.  **Important Considerations:**  * **Patient Factors:** The optimal treatment plan will depend on various factors, including the patient's age, overall health status, prior treatments, and treatment goals.  * **Side Effects:** All treatment options have potential side effects. It's crucial to discuss the risks and benefits of each treatment with the oncologist.  * **Supportive Care:** Adequate supportive care, including management of treatment side effects, is essential throughout the course of treatment.  **Recommendations:**  * **Thorough Discussion:** The oncologist should have a detailed discussion with the patient to understand their medical history, treatment goals, and preferences.  * **Multidisciplinary Team:** A multidisciplinary team approach, involving medical oncologists, surgeons, pathologists, and other specialists, is often beneficial in managing metastatic breast cancer.  * **Regular Monitoring:** Close monitoring of the patient's condition, including regular tumor assessments and blood tests, is crucial to assess treatment response and adjust the treatment plan as needed.  **Disclaimer:** This information is for general knowledge and discussion purposes only. It does not constitute medical advice. The treatment of metastatic breast cancer is complex and should always be overseen by a qualified oncologist.  This information is for general knowledge and discussion purposes only. It does not constitute medical advice. The treatment of metastatic breast cancer is complex and should always be overseen by a qualified oncologist. | | | | | | | |

| **MTB-Like Scenario Example – Google Gemini 1.5 Flash** | | | | | | | |
| --- | --- | --- | --- | --- | --- | --- | --- |
| **Age** | **Disease Type/Stage/Metastasis** | **Prior Treatments** | **Mutations** | **Rater #** | **Accuracy** | **Completeness** | **Confidence** |
| 60 | MSI-High HER2 non-amplified Gastric Cancer/NA/NA | Pembrolizumab | TP53 P152Rfs*18, PIK3CA E545K, BRCA1 E1250K, ARID1A D1850Tfs*33 | **1** | 2 | 2 | 2 |
|  |  |  |  | **2** | 3 | 3 | 3 |
|  |  |  |  | **3** | 3 | 2 | 3 |
| **Example of Output:** | | | | | | | |
| This is for informational purposes only. For medical advice or diagnosis, consult a professional.  **1. Treatments Suggested by Molecular/Genetic Testing:**  * **PIK3CA Mutation:**  * **Alpelisib (Piqray):** While not specifically approved for gastric cancer, alpelisib is an FDA-approved PI3K inhibitor for postmenopausal women with hormone receptor-positive, HER2-negative breast cancer with PIK3CA mutations. Its mechanism of action suggests potential activity in gastric cancer with PIK3CA mutations, especially considering the patient's progression on pembrolizumab.  * **BRCA1 Mutation:**  * **PARP Inhibitors:** The presence of a BRCA1 mutation may increase sensitivity to PARP inhibitors like olaparib, rucaparib, and talazoparib. These drugs exploit DNA repair deficiencies in BRCA-mutated tumors.  **2. Additional Treatment Options:**  * **Chemotherapy:**  * **Second-line chemotherapy options:** Depending on prior therapies, options may include irinotecan-based regimens (e.g., FOLFIRI), oxaliplatin-based regimens (e.g., FOLFOX), or other combinations.  * **Immunotherapy:**  * **Alternative ICIs:** If pembrolizumab has failed, exploring other ICIs like atezolizumab or nivolumab in combination with ipilimumab might be considered.  * **Clinical Trials:**  * Participation in clinical trials investigating novel therapies for MSI-high gastric cancer with these specific mutations is strongly encouraged. These trials may offer access to the latest and most promising treatments, including combinations of PI3K inhibitors, PARP inhibitors, and other targeted therapies.  **Important Considerations:**  * **Patient Factors:** The optimal treatment plan will depend on various factors, including the patient's age, overall health status, prior treatments, and treatment goals.  * **Side Effects:** All treatment options have potential side effects. It's crucial to discuss the risks and benefits of each treatment with the oncologist.  * **Supportive Care:** Adequate supportive care, including management of treatment side effects, is essential throughout the course of treatment.  **Recommendations:**  * **Thorough Discussion:** The oncologist should have a detailed discussion with the patient to understand their medical history, treatment goals, and preferences.  * **Multidisciplinary Team:** A multidisciplinary team approach, involving medical oncologists, gastroenterologists, radiologists, and other specialists, is often beneficial in managing advanced gastric cancer.  * **Regular Monitoring:** Close monitoring of the patient's condition, including regular tumor assessments and blood tests, is crucial to assess treatment response and adjust the treatment plan as needed.  **Disclaimer:** This information is for general knowledge and discussion purposes only. It does not constitute medical advice. The treatment of advanced gastric cancer is complex and should always be overseen by a qualified oncologist.  **Note:** The use of alpelisib and PARP inhibitors in gastric cancer with these specific mutations is considered investigational.  This information is for general knowledge and discussion purposes only. It does not constitute medical advice. The treatment of advanced gastric cancer is complex and should always be overseen by a qualified oncologist. | | | | | | | |
